# The effect of 10 weeks of karate training on the development of motor skills in children who are new to karate

**DOI:** 10.3389/fphys.2024.1347403

**Published:** 2024-04-02

**Authors:** Yasin Arslan, Belma Yavaşoğlu, Ayşegül Beykumül, Aylin Özge Pekel, Ceren Suveren, Ebru Olcay Karabulut, Tebessüm Ayyıldız Durhan, Veli Ozan Çakır, Nuriye Sarıakçalı, Hamza Küçük, Levent Ceylan

**Affiliations:** ^1^ Faculty of Sports Sciences, Gazi University, Ankara, Türkiye; ^2^ Health Sciences Institute, Kırşehir Ahi Evran University, Kırşehir, Türkiye; ^3^ Turgut Özal Medical Center, İnönü University, Malatya, Türkiye; ^4^ Institute of Social Sciences, Sivas Cumhuriyet University, Sivas, Türkiye; ^5^ Yaşar Doğu Faculty of Sports Sciences, Ondokuz Mayıs University, Samsun, Türkiye; ^6^ Faculty of Sports Sciences, Hitit University, Çorum, Türkiye

**Keywords:** karate, motor development, Eurofit test battery, TGMD-2, children

## Abstract

This study investigated the effect of a 10-week karate training program on the motor skill development of 5-7-year-old children new to karate with two different test batteries. A total of 28 participants were included in the study: 18 in the Karate group and 10 in the control group. The karate group was subjected to a fundamental karate training (kihon) program consisting of 90-minute sessions four days a week for ten weeks in addition to physical education classes at their schools. In contrast, the control group did not participate in any sportive activities except physical education classes in their schools. Data were collected using the Eurofit test battery and the TGMD-2 test. In the pre-post test comparison of the anthropometric measurements of the karate group, no significant difference was found in the control group. In contrast, a significant difference was obtained in height, body mass index, and body fat percentage. In the post-test analysis of the two independent groups, there were statistically significant differences in favor of the karate group regarding height and body fat percentage (*p* < 0.005). In the pre-post analysis of the Eurofit test and the TGMD-2 for the karate group, all parameters showed statistically significant improvements (*p* < 0.001), while the control group showed no statistical difference. After comparing the karate and control groups, the Eurofit Test and TGMD-2 post-test results showed significantly higher scores (statistically significant differences) in all parameters for the karate group. In conclusion, the study shows that the 10-week karate training program positively affected the motor development of the participating children.

## 1 Introduction

The foundations of development are laid in the mother’s womb. In this context, it is undeniable that the healthier the developmental periods of children from this period, the healthier they will be in the future. In his theory of motor development, Gallahue examined motor development in four periods. These periods are the period of reflexive movements, the period of primitive movements, the period of basic movements, and the period of sports movements (Skowroński et al., 2018). Preschool and school periods are the periods of basic movements of children. This period, which is the next step after reflexive and primitive movements in infancy, plays a decisive role in the movement capacity of individuals in adulthood. Children develop basic skills such as locomotor and object control during this period. Children who develop these skills have formed the primary acquisition of the sports life they will continue in the future, or when they become adults, they lay the foundation of the movement capacity that will accompany them throughout their lives. Therefore, for a better quality of life, every child should play sports for development, not just for success, even if it is not at a very high level. Sports practiced from an early age positively support children’s physical, cognitive, social, and spiritual development (Çelik and Şahin, 2013; Pamuk et al., 2023).

The Japanese Karate-Do discipline is a Far Eastern combat sport based on using unarmed self-defense. Contrary to popular belief, it was developed for defense, not attack. Karate has two sub-branches, kata and kumite (Alpay, 2016; Çetintaş and Yavuz, 2021; Dilekçi, U, 2021).

To reach a certain level in any sport, it is necessary to have motor competence. In this sport, which requires specialization at an early age, considering that athletes participate in competitions from an early age and are competitive athletes, the physical fitness and motor development of children engaged in this sport become very important. Physical activity and sports play an influential role in individuals’ sociological, psychological, and mental development. Many studies have shown that the health and performance-specific physical fitness components of individuals participating in sporting activities improve more than those who are sedentary (Barnett et al., 2008; Cho, 2020; Erkek et al., 2022).

Children need continuous exercise during their growth and development. Many studies have shown that inactivity in childhood leads to health problems later in life (Telama et al., 2014; Cho, 2020). Therefore, the importance of movement development programs implemented in this period is emphasized by many researchers (Suveren-Erdoğan, 2014; Shen et al., 2020). Movement skills acquired in the first few years of life and during childhood prepare the ground for more complex movement skills expected to be realized later in life (Suveren-Erdoğan, 2014).

Karate is a sport that positively impacts children’s physical fitness and motor skills (Alesi et al., 2014; Boguszewski and Socha, 2011; Ma and Qu, 2017; Rutkowski et al., 2019). Because it focuses on developing intermuscular coordination by using the upper and lower extremities at the same time, balance by paying attention to maintaining body position in static and dynamic situations, reaction speed to react quickly to attacks or poses, muscular strength for effective strikes, and endurance to keep performance for a certain period (Alesi et al., 2014; Ma and Qu, 2017). In addition to its impact on physical and motor skill development, karate contributes significantly to children’s mental, social, and psychological development (Alesi et al., 2014; Moore et al., 2019). Practicing karate at an early age can raise healthy individuals and potential elite athletes in the future as it is compatible with the primary movement phase, a critical period in motor development.

Karate techniques usually require quickness and strength. Karate competitions take place in an 8 × 8 square area. Within this area, there is a need to quickly overcome and defeat another intelligence with a fast game intelligence. Therefore, a short reaction time is significant. Performing movements in a small area increases the importance of speed and quickness for karate players. Flexibility is one of the most essential criteria for correctly applying karate techniques that require a wide range of movement. A wide range of motion is needed to punch farther, kick higher, and move comfortably in the field (Turgut, 2016). The hypothesis underlying this research assumes that karate positively affects young children’s motor development. This hypothesis is confirmed by the convergence and remarkable similarity of the results obtained from two different battery tests.

Given the paucity of existing research investigating the impact of karate training on children’s developmental outcomes, this study is expected to contribute to the field significantly. There needs to be more studies in the literature that specifically analyze the impact of training stimuli through karate on children’s motor development by assessing it through multiple methods and then making a discourse on the relevance of these findings. For these reasons, our research is considered innovative as it presents an original investigation.

The primary purpose of this research study is to determine whether karate practice has a positive effect of 5-7-year-old individuals who are new to this discipline. We hypothesize that it will have a positive impact. We approached this research by using two sets of assessment protocols and focusing on the consistency of the results obtained from these other assessment methods.

## 2 Material and methods

### 2.1 Research group

The study sample consisted of 28 children aged 5-7 who received preschool and study education at Şefkat Dünyası Kindergarten and Study Center and had just started karate training at Reykan Sports Club. When the literature was examined, it was seen that the motor performance of pre-adolescent children increased with age. Still, there was no significant difference in motor performance when gender was considered. Therefore, the study’s results were not analyzed according to gender variables (Cho, 2020; Pekel, 2023; Suggate et al., 2017; Yavuz et al., 2021).

Children who had just started karate at Reykan Sports Club constituted the karate group of the study (18 participants), and children who received only preschool and study education included the study’s control group (10 participants). Ethical approval was obtained from the Gazi University Ethics Committee for this study, and the necessary permissions were obtained by sending the participant information and voluntary consent form to the parents of the children. Boys and girls aged 5–7 years who had never practiced sports before, had no health problems or disabilities, participated in this study voluntarily, participated in both pre and post-measurements and continued to train regularly were included in the karate group. The karate group received 30 min of warm-up and 60 min of karate training 4 days a week for 10 weeks on the days and times determined by the sports club for this age group. The karate group received basic karate training called “kihon.” Participants in the control group did not participate in any sports activity.

### 2.2 Data collection tools

Data were collected using anthropometric measurement tests, Eurofit test battery, and TGMD-II tests. TGMD-II consists of two subtests. Basic motor and object control skills were tested. The tests of fundamental motor skills included running, galloping, jumping, skipping, jumping, horizontal jumping, and sliding. In contrast, the tests of object manipulation skills included kicking a stationary ball, dribbling a stationary ball, catching, kicking, overhead throwing, and underhand rolling. Measurements were performed in two repetitions for all children. Each test has more than one evaluation criterion, and for each criterion, raw data were obtained by assigning 1 point to successful skills and 0 points to unsuccessful skills (Marshall and Bouffard, 1997; Ulrich, 2000; Penedo and Dahn, 2005; Yıldırım, 2011; Kerkez, 2013; Kim et al., 2014). The Eurofit test includes flamingo balance, plate hit, sit-stand, standing long jump, handgrip, sit-up, bent arm hang, and 20-meter sprint tests (EUROFIT, 1993).

The assessment measurements for the karate and control groups were performed simultaneously on different days and at the same time. This strategic approach ensured that the two groups were assessed under similar conditions. To avoid a possible bias, one test was administered in the morning and the other in the afternoon, with a sufficient rest period in between. This deliberate interval was used to ensure that participants had enough time to recover and to eliminate the effect of fatigue or any immediate effects from the previous assessment.

### 2.3 Data collection

Anthropometric Measurements: Height: Participants’ height was measured with a German “Seca” stadiometer with an accuracy of ±0.1 cm. With the head in the frontal plane, body weight was distributed equally between both feet, and measurements were taken with bare feet and heels in contact with the stadiometer (Marangoz and Koç, 2021).

Body Weight and Body Fat Percentage: Participants’ body weight and body fat percentage were measured barefoot in sportswear with an accuracy of ±0.1 kg using a “Tanita” brand body composition analyzer developed in Japan (Cho, 2020).

Body Mass Index: Body weight (kg)/height^2^ (m). The participant’s body mass index was calculated as the ratio of their body weight in kilograms to the square of their height in meters (Sarría et al., 2001).

Eurofit Test Battery: The Eurofit test includes flamingo balance, plate tapping, sit-stand, standing long jump, handgrip, sit-up, bent arm hang, and 20-meter sprint tests (EUROFIT, 1993).

TGMD-II Tests: TGMD-II consists of two subtests. Primary motor and object control skills were tested. The tests of basic motor skills included running, galloping, jumping, skipping, jumping, horizontal jumping, and sliding. In contrast, the tests of object manipulation skills included kicking a stationary ball, dribbling a stationary ball, catching, kicking, overhead throwing, and underhand rolling. The measurements were performed in two repetitions for all children, and 1 point was given for successful skills and 0 points for unsuccessful skills (Penedo and Dahn, 2005; Yıldırım, 2011).

### 2.4 Data analysis

SPSS 24 was used for data analysis. ANCOVA analysis was used to determine whether the groups differed significantly. The compared groups were included as indicator variables in the model. The t-test was used to test the difference between the karate group and the control group in terms of anthropometric variables.

## 3 Results

The results of our study are illustrated in Tables 1-3.

**TABLE 1:** ANCOVA Analysis of pre/post-test anthropometric scores for study and control groups.

Anthropometric Measurements	Group	Pre		Post		*F*	*p*	Partial eta
		*x*	sd	*x*	sd			
Height (cm)	Karate Group	120.57	9.15	125.05	9.74	5.395	0.003	0.237
	Control Group	113.11	8.48	113.42	8.59			
Body weight (kg)	Karate Group	23.87	5.36	23.71	4.94	1.916	0.138	0.100
	Control Group	20.68	3.66	20.65	4.79			
Body Mass Index (kg/m^2^)	Control Group	16.26	2.18	15.03	1.63	1.509	0.223	0.080
	Karate Group	16.09	1.84	15.97	15.79			
Body Fat Percentage	Control Group	19.50	4.63	15.1	3.03	6.273	0.001	0.266
	Karate Group	19.33	2.27	19.33	2.27			

When the post-test values of anthropometric measurements of the karate and control groups were compared, a significant difference was found in favor of the karate group regarding height and body fat percentage (*p* = 0.003; *p* = 0.001) in table.

**TABLE 2:** ANCOVA Analysis results of eurofit pre-post test parameters for karate and control groups.

	Group	Pre		Post		*F*	*p*	Partial eta
		Mean	sd	Mean	sd			
Right Static Balance	Karate Group	31.56	5.50	12.94	3.52	49.991	<0.001	0.743
	Control Group	31.60	6.33	32.70	6.95			
Left Static Balance	Karate Group	31.00	5.81	14.00	2.91	47.798	<0.001	0.734
	Control Group	31.50	5.84	31.30	5.66			
Plate Tapping (sec)	Karate Group	35.48	5.46	23.16	5.89	33.410	<0.001	0.658
	Control Group	38.91	3.43	38.61	3.34			
Sit-Reach (cm)	Karate Group	15.69	2.19	25.62	3.02	57.160	<0.001	0.767
	Control Group	15.84	2.66	15.66	2.63			
Standing Long Jump (cm)	Karate Group	87.42	16.47	117.68	11.75	17.773	<0.001	0.506
	Control Group	85.68	15.28	57.98	15.05			
Right Hand Grip	Karate Group	8.60	2.15	11.55	2.40	6.488	<0.001	0.272
	Control Group	8.93	2.04	8.45	2.65			
Left Hand Grip	Karate Group	8.60	1.96	11.46	2.27	7.963	<0.001	0.315
	Control Group	8.72	1.89	8.20	2.28			
Sit-Up	Karate Group	9.83	2.33	18.44	3.36	41.265	<0.001	0.704
	Control Group	9.00	2.71	9.00	8.83			
Bent Arm Hanging (sec)	Karate Group	3.55	1.11	8.02	2.63	27.039	<0.001	0.609
	Control Group	3.43	1.17	3.36	1.11			
Shuttle Run	Karate Group	7.39	1.58	15.00	2.79	68.316	<0.001	0.798
	Control Group	6.30	1.95	5.30	1.49			

A significant difference (*p* < 0.01) was found between the pre and post-test scores of the Eurofit test battery for all parameters in the karate group. No significant difference was found when the pre and post-test values of the Eurofit test parameters of the control group were compared in table.

**TABLE 3:** ANCOVA Analysis results of tGMD-2 pre-post test parameters for karate and control groups.

TGMD-2 Parameters	Group	Pre-test		Post-test		*F*	*p*	Partial eta
		x	sd	x	sd			
Locomotor Skills	Karete Group (n = 18)	14.11	3.25	39.33	2.67	328.71	<0.001	0.950
	Control Groups (n = 10)	12.90	2.46	13.10	2.84			
Object Control Skills	Karete Group (n = 18)	11.27	1.70	38.16	2.47	712.38	<0.001	0.976
	Control Groups (n = 10)	10.60	1.95	10.90	1.85			

A significant difference was observed in all parameters in the TGMD-2 pre/post comparison of the karate (*p* < 0.001). No significant difference was found in the TGMD-2 pre/post-test comparison of the control group. No significant difference was found when the TGMD-2 pre-test parameters of the karate and control groups were compared. When the TGMD-2 post-test parameters of the karate and control groups were compared, a significant difference was observed in favor of the karate group for all parameters in table.

## 4 Discussion and conclusion

The TGMD-2 and Eurofit Test Battery are among the tests commonly used to assess children’s motor development. Early assessment of children’s development helps to identify deficits in motor development and to design appropriate programs to improve motor skills. To prepare educational programs to enhance children’s motor performance, it is necessary to know the level of motor development (Goodway et al., 2019; Kerkez, 2013; Suveren-Erdoğan, 2014).

In the pre/post comparison of anthropometric measurements of the karate and control groups were compared, a significant difference was found in favor of the karate group regarding height and body fat percentage (*p* = 0.003; *p* = 0.001) in Table 1. The significance seen in the pre/post-test comparison in the karate group and in the anthropometric measurement post-test parameters in which the two groups were compared may be associated with the support of the developmental processes of the children participating in the study with karate training. The significance observed in the pre/post-test comparison of the control group and the height parameter in the anthropometric measurement pre-test in which the two groups were compared may be associated with the general developmental processes of the children who voluntarily participated in the study.

In a study conducted on male athletes, Koç and Gökdemir observed significant increases in height and body weight parameters (Koç and Gökdemir, 1997). In parallel with Koç and Gökdemir’s study, other studies have also reported significant height and body weight increases in the athlete and non-athlete groups. The height increase continued with age (Bale et al., 1992; Fjørtoft, 2000; Houwen et al., 2006; Kalkavan, 1999; H. A. Pekel et al., 2006). Mengütay stated that these significant increases in children’s height and weight are due to development during childhood and adolescence (Božanić and Bešlija, 2010).

In a study conducted by Boz and Aytar in 2012, they gave basic movement training to children aged 5–6 years for 12 weeks and examined the effect of this training on the development of movement skills. As a result of the research, they concluded that the movement skills of the group receiving basic movement training improved positively (Goodway et al., 2019). In their study conducted in 2016, Demirel et al. examined the physical development of children aged 3–6 years who practiced gymnastics using the Eurofit Test Battery. As a result of the research, they saw that the gymnastics group improved in some Eurofit parameters (Demirel et al., 2016). In this study, in which basic karate training was applied for 10 weeks to children aged 5–7 years for a similar duration and age group, it was concluded that movement skills developed positively with two different test batteries in Tables 2, 3.

In their 2010 study, Bozanic and Beslija examined the relationship between these special technical skills and basic movement skills, stating that children who start karate receive basic karate training, “kihon” training, at the beginning of their sports career and that this training includes special skills such as hand and foot strikes and blocking. They used the TGMD-2 to assess motor skills. As a result of the research, they concluded that karate-specific basic technical skills and basic motor skills were significantly related. They observed that children with basic motor skills had better karate techniques (Božanić and Bešlija, 2010). Based on the positive effect of basic motor skills on karate technique, this study investigated whether karate training affects basic motor skills. As a result of this research, it was seen that kihon training, which is the same training program, positively affects basic motor skills.

In a study conducted in 2019, Rutkowski et al. gave 10 weeks of karate training to school-age children. They examined the changes in their physical fitness using the Eurofit test battery. As a result of the research, they found that the physical fitness of the group given karate training improved positively (Rutkowski et al., 2019). In their study in 2021, they looked at the development of motor skills using the Eurofit test battery by having school-age children do core and karate training. As a result of the research, they reported that school-age children practicing karate showed positive improvements in some motor skill parameters measured with the Eurofit Test Battery (Chovanova et al., 2021). In a study conducted by Boguszewski and Socha in 2011, the effects of karate training on the physical fitness and motor development of boys and girls aged 4.5–6.5 years were investigated. The findings of their study revealed remarkable results. Among the male participants who practiced karate, the most impressive improvements were recorded in flexibility, lower limbs, and abdominal muscle strength. In contrast, female participants showed the most remarkable improvements in all assessments. This study concluded that karate training positively affects preschool children’s motor development (Boguszewski and Socha, 2011).

Similarly, Alesi et al. initiated a study in 2014 investigating the impact of karate on cognitive and motor development. Their findings showed that children who participated in karate showed improved performance in speed-based tasks, explosive power assessments, and coordination assessments.

The study also revealed that karate positively affected children’s physiological and psychological development (Alesi et al., 2014). In parallel, Ma and Qu 2017 investigated the effects of karate training on motor development by giving karate training to primary school children with no previous experience in this discipline. The findings showed significant differences between the participants in medicine ball throwing, 4 × 5 m shuttle test, and long jump. These results underlined the positive effect of karate training on the motor development of elementary school children who were new to karate (Ma and Qu, 2017).

This study is in accordance with abovementioned studies. According to the results of this study, statistically significant results were obtained in favor of the karate group in all parameters in Eurofit Test and TGMD-2 pre-post test comparisons (*p* < 0.001). On the other hand, no statistically significant difference was found in favor of the control group in both test batteries in pre-post test comparisons. Eurofit test and TGMD-2 posttest comparisons of karate and control groups showed statistically significant results in favor of the karate group for all parameters. It was concluded that these effective motor development results obtained with both test batteries were due to the karate training applied to the karate group.

In the literature review, no other study was found that examined the effect of karate training on motor development using TGMD-2 and Eurofit Test Battery as in this study. In this respect, this study is an original research. In the literature, the Eurofit Test Battery is generally used to investigate the effect of training stimulus on children’s motor development. In contrast, TGMD-2 is used to determine the motor development level of children, to evaluate whether the developmental scale increases with age or to investigate motor development differences between sports (Alesi et al., 2014; Marshall and Bouffard, 1997; Penedo and Dahn, 2005; Rutkowski et al., 2019; Yıldırım, 2011). However, unlike the literature, this study was conducted to determine the effect of the training stimulus given with TGMD-2 on motor development and to examine the similarity of this effect with the Eurofit Test Battery. As a result of the study, it was determined that the results of the Eurofit Test Battery and TGMD-2 were in parallel with each other.

The study started with 33 children: 15 in the control group and 18 in the karate group. However, five athletes could not participate in the final measurements for personal reasons. Therefore, the control group was reduced to 10, and the study was completed with 28 participants.

## 5 Conclusion

The results show that ten weeks of karate training had a significant effect on both the TGMD-2 and the Eurofit test battery. These findings are important for educators and coaches as they provide valuable information for curriculum development and physical education strategies, emphasising the potential of karate training to improve overall fitness.

### 5.1 Limitation

The study started with 33 children: 15 in the control group and 18 in the karate group. However, five athletes could not participate in the final measurements for personal reasons. Therefore, the control group was reduced to 10, and the study was completed with 28 participants.

## Data Availability

The raw data supporting the conclusion of this article are provided by the authors without reservation.
